# Influence of Different Types of Drying Methods on Color Properties, Phenolic Metabolites and Bioactivities of Pumpkin Leaves of var. Butternut squash (*Cucurbita moschata* Duchesne ex Poir)

**DOI:** 10.3389/fnut.2021.694649

**Published:** 2021-06-29

**Authors:** Florence M. Mashitoa, Tinotenda Shoko, Jerry L. Shai, Retha M. Slabbert, Yasmina Sultanbawa, Dharini Sivakumar

**Affiliations:** ^1^Phytochemical Food Network Research Group, Department of Crop Sciences, Tshwane University of Technology, Pretoria West Campus, Pretoria, South Africa; ^2^Department of Horticulture, Tshwane University of Technology, Pretoria West Campus, Pretoria, South Africa; ^3^Department of Biomedical Sciences, Tshwane University of Technology, Acadia Campus, Pretoria, South Africa; ^4^ARC Training Centre for Uniquely Australian Foods, Centre for Nutrition and Food Sciences, Queensland Alliance for Agriculture and Food Innovation, The University of Queensland, Coopers Plains, QLD, Australia

**Keywords:** Cucurbitaceae, polyphenols, α-glucosidase, FRAP activity, chlorophyll

## Abstract

Leaves of pumpkin species var. Butternut squash (*Cucurbita moschata* Duchesne ex Poir) is a popularly consumed leafy vegetable in the Southern African region. Traditional vegetables are commonly sun-dried as a method of postharvest preservation during the off-season. However, different drying methods affect the superior quality, functional properties, and bioactivities of the final product. Therefore, in this study, var. Butternut squash (*C. moschata*) underwent different drying methods, such as freeze-, oven, sun-, solar, and microwave drying to evaluate the color properties, pigments, phenolic metabolites, *in vitro* antioxidants, and antidiabetic activities. Results indicate that freeze-drying retained the total chlorophyll content with green color by reducing the color difference *(*Δ*E*), improved the concentration of different phenolic metabolites and the content of ascorbic acid, and enhanced the FRAP, ABTS activities and the inhibitory effects of α-glucosidase, and α-amylase. Freeze-dried leaves contained the highest concentrations of quercetin 3-glucoside 7-rhamnoside (rutin), quercetin 3-galactoside, isorhamnetin-3-galactoside-6″-rhamnoside, isorhamnetin-3-O-rutinoside compared with the leaves that underwent four other drying treatments and raw leaves. The OPLS-DA and the UPLC–QTOF/MS and chemometric approach showed that the peak at *m*/z 609, 1441 (quercetin 3-galactoside 7-rhamnoside) separated the freeze-dried leaves of var. Butternut squash (*C. moschata*) from the other four drying treatments. Therefore, freeze-drying is highly recommended to obtain good quality leaf powders that are rich in functional compounds and bioactive properties for use as functional ingredients.

## Introduction

Pumpkin species var. Butternut squash (*Cucurbita moschata* Duchesne ex Poir), belonging to the family Cucurbitaceae, is indigenous to Mexico and Central America and is considered as an indigenous vegetable in the African region due to its naturalization in Africa (1). The FAO/WHO (2) recommends a serving size of 80–100-g green leafy vegetables in four to five servings for adults. The nutritional information present in the leaves of pumpkin species *C. moschata* is given in detail in the study of Mashitoa et al. (3). The leaves of pumpkin species (*C. moschata*) are functional vegetables due to their higher phenolics, flavonoid content, and antioxidant scavenging activity (4–6). In addition, leaves of pumpkin species (*C. moschata*) contain cinnamic acid, *p*-hydroxybenzoic acid, gentisic acid, protocatechuic acid, *p*-coumaric acid, ferulic acid, and caffeic acid (4). Ko et al. (4) reported the presence of caffeic acid, *p*-coumaric acid, ferulic acid, and gentisic acid as the predominant phenolic compounds in the leaves of pumpkin species (*C. moschata*). Pumpkin leaves are highly perishable, and the lack of cold storage infrastructure in rural areas affects their marketability. Therefore, postharvest drying is a suitable way to reduce losses and increase shelf life by preventing microbial decay without adding preservatives. Currently, different types of drying systems, such as solar drying, freeze-drying, oven drying, and microwave drying, are available.

Solar drying uses renewable energy, which is cost effective and easy to operate. Bananas, pineapples, mangoes, and vegetables, such as, potatoes and carrots, are currently solar dried (7). Oven drying is a common method used but is energy inefficient (8). Conventional microwave drying adds benefits to fresh produce such as fruits that have higher sugar content and improves their quality (9). Furthermore, freeze-drying works based on the sublimation process and helps to preserve the biochemical constituents due to the temperatures involved (as low as −50 to −80°C) by stopping the changes (10).

Managa et al. (11) reported the traditionally adopted sun-drying method negatively affected the product quality due to changes in phytonutritional compounds and the lack of food safety guidelines. Besides, the demand for dried vegetable powders, known as the functional powders demand, are increasing for use as nutritional and fortifying food additives. The functional foods market size will increase to US$275.77 in 2025 due to the increasing consumer demand for healthy food (12). However, temperature and duration of drying are vital since they affect the chemical, functional properties, and quality of the dried food product; thus, the selection of the best drying method depends on quality requirement and the affordability of the drying method (13). Therefore, the objective of this study was to investigate the influence of different drying methods, such as drying in a solar cabinet dryer, a hot air oven, freeze-drying, and traditional sun-drying methods, on the changes in (i) color properties, (ii) changes in lipophilic pigments, (iii) phenolic metabolite components, (iv) ascorbic acid, and (v) antioxidant property of the leaves of var. Butternut squash pumpkin (*C. moschata*).

## Materials and Methods

### Pumpkin Leaves

Leaves of var. Butternut squash (*C. moschata*) were harvested from the Tshiombo irrigation scheme in Venda, Limpopo, South Africa, in the summer of 2019. The harvesting of pumpkin leaves took place 3–4 weeks after planting, and leaves that were free from dirt, pest damage, or decay were selectively harvested and washed in tap water, as described previously by Managa et al. (11).

### Chemicals

All chemicals used in this study were purchased from Sigma Aldrich, Johannesburg, South Africa.

### Postharvest Drying Treatments

This study adopted four different drying methods as follows:

Solar drying, using a solar dryer erected for the Tshiombo tribal council community jointly with Scaled Impact Pty (Ltd), Johannesburg and Tshwane University of Technology, Pretoria, South Africa, as described previously by Managa et al. (11). In the solar cabinet, uniform size leaves (380 g) were placed in 20 trays (894 × 605 × 80 mm), and the warm air circulated uniformly through the trays. The air circulation was facilitated with fans fitted with the solar photovoltaic panel. The solar cabinet was set at 45–50°C and the RH to around 30–45% based on the weather conditions. Drying duration on a warm day was 48 h and was continued until the leaves obtained constant weight.

Oven drying was carried out by placing 5–6 kg of the samples of leaves of var. Butternut squash (*C. moschata*) on a stainless-steel tray (915 × 840 × 915 mm) and drying them in a conventional drying oven (95 L) with Forced Convection System (Digital series, EcoTherm, Hartkirchen, Austria) at 100°C, RH 12% to 14% until constant weight (2 days), as described previously by Managa et al. (11).

Freeze-drying, pumpkin leaves (5–6 kg) were frozen at −80°C and then freeze-dried in a vacuum freeze-drier (YK-118-50, Taiwan) between −47 and −53°C for 72 h, as described previously by Managa et al. (11).

Microwave drying was performed in a domestic digital microwave oven (Samsung 40l microwave oven, Seoul, South Korea) with 230 V, 50 Hz, a frequency of 2,450 MHz (a wavelength of 12.24 cm), and an output power of 900 W for 15 min using 25 g of a leaf sample spread uniformly on the rotating glass platform of the microwave (11).

Sun-drying was performed by placing 5–6 kg of the samples of leaves of var. Butternut squash (*C. moschata*) on the pumpkin leaves on plain white paper sheets and under direct sun for 2 days to mimic the traditional way of drying adopted by the rural communities.

### Color Changes

Dried leaves of var. Butternut squash (*C. moschata*) leaves were ground into a homogeneous powder using a domestic coffee grinder for 30 s, as described by Managa et al. (11). Color coordinates, L^*^, a^*^, *b*, were recorded with a color meter (CM-700d, Konica Minolta Sensing Inc., Tokyo, Japan), as described previously by Managa et al. (11), and the total color change, Δ*E*, was calculated to show the color difference between fresh (raw) and dried leaves.

### Color Pigments

Leaf pigments, chlorophyll a (*Chl a*) and b (*Chl b*), total chlorophyll, and carotenoid concentrations were quantified byadopting the method described by Mampholo et al. (14) and Managa et al. (11). Both chlorophyll a (*Chl a*) and b (*Chl b*) and total chlorophyll were determined using pumpkin leaf samples (0.2 g), homogenized with 2-ml acetone and hexane 4:6 (v/v), thereafter extracted for 2 h, and centrifuged (Hermle Labortechnik GmbH, Z326 K, 2010, Wehingen, Germany) for 10 min at 4°C (9,558 × g). The resulting supernatant was decanted and a portion of the solution was measured at 470, 646, and 662 nm (Biochrom Anthos Zenyth 200 Microplate Reader; SMM Instruments, Biochrom Ltd., Johannesburg, South Africa). The *Chl a* and *Chl b* contents were determined according to Managa et al. (11) using the following equations: *Chl a* = 15.65A662 – 7.340 A646, *Chl b* = 27.05 A646 −11.21 A662. The total chlorophyll was determined from *Chl a* + *Chl b*. Total carotenoids = (1,000 A470 – 2.270 *Chl a* – 81.4 *Chlb*)/227. All pigments were expressed in mg per 100 g on a dry-weight basis.

### Ascorbic Acid Content

Ascorbic acid content was quantified using 30 g of leaves of var. Butternut squash (*C. moschata*) with five replicates from each drying treatment, according to Managa et al. (11) without any modifications, adopting the 2,6-dichlorophenol-indophenol titration method. A set of 5 g of leaves was homogenized in 10 ml of 4% oxalic acid, and then the mixture was filtered; the final volume was converted to 25 ml using 4% oxalic acid and quantified in 100 g^−1^ mg on a dry-weight basis.

### Changes in Phenolic Metabolite Profile and Multivariate Analysis

Predominant polyphenolic metabolites present in leaves of var. Butternut squash (*C. moschata)* were extracted and quantified using a Waters Synapt G2 Quadrupole time-of-flight (QTOF) mass spectrometer (MS), hyphenated to a Waters Aquity ultra-performance liquid chromatograph (UPLC) (Waters, Milford, MA, USA), as described earlier by Mashitoa et al. (3). Briefly, the instrument was used in the negative mode electrospray ionization with a cone voltage of 15 V, a desolvation temperature of 275°C, and desolvation gas of 650 L/h, and the rest of the MS settings were optimized for best resolution and sensitivity. Data were obtained by scanning from 150–1,500 *m/z* in the resolution mode as well as in the MSE mode. Two channels of MS data were acquired in the MS mode, one at low collision energy (4 V) and the second using a collision energy ramp (40–100 V) to obtain fragmentation data. Leucine enkephalin was used as a lock mass (a reference mass) for accurate mass determination, and the instrument was calibrated with sodium formate. Separation was achieved on a Waters HSS T3, 2.1 × 100 mm, a 1.7-μm column. The injection volume was 2 μl with the mobile phases being water with 0.1% formic acid (solvent A) and acetonitrile containing 0.1% formic acid as solvent B. The following chromatographic method was used on 100% of solvent A for 1 min, changing to 28% of solvent B over 22 min then 40% of solvent B over 50 s, and a wash of 1.5 min at 100% of solvent B was carried out. The final step was re-equilibration to initial conditions for 4 min. The flow rate was 0.3-ml min^−1^, and the column temperature was 55°C throughout the run. Three replicate samples of 50 mg from oven drying, solar drying, microwave drying, and freeze-drying were prepared by ultrasonication in 70% aqueous ethanol. The different phenolic components were quantified, using the reference calibrants catechin (LOD 1.41, LOQ 4.29), epicatechin (LOD 5.11, LOQ 15.5), and rutin (LOD 3, 29; LOQ 9.98), based on the areas of their extracted mass chromatograms. Thereafter, the obtained data were processed using TargetLynx software, as described previously by Mashitoa et al. (3), and the concentration of phenolic compounds was expressed as mg kg^−1^. The differences between the phenolic metabolic profiles of the different postharvest drying treatments were analyzed, using an unsupervised Principal Component Analysis (PCA) approach using the data obtained from the UPLC–Q-TOF/MS. PCA was conducted to reduce the number of variables in the data matrix in order to choose the most discriminating postharvest drying on the phenolic metabolites in the leaves of var. Butternut squash *C. moschata* (3). Therefore, UPLC data were exported as an mzXML file, aligned by Marker Lynx 4.1 in the Apex Trac™ tool for the PCA analysis. However, to explain the differences between the different kinds of postharvest drying and to identify the potential characteristic markers (compounds) responsible for discrimination between the different postharvest drying treatments, supervised Orthogonal Projections to Latent Structures Discriminant Analysis (OPLS-DA) was undertaken.

### Total Antioxidant Capacities

The ferric-reducing antioxidant power (FRAP) assay was performed according to the method of Managa et al. (11) and Mashitoa et al. (3) for leaves of var. Butternut squash (*C. moschata*). As described previously by Mashitoa et al. (3), leaf samples (0.20 g) were homogenized in 2 ml of 80% aqueous methanol. A 15-μl aliquot of this leaf extract was incubated for 10 min with 220 μl of the FRAP reagent solution [10 mmol L^−1^ TPTZ [2,4,6-tris(2-pyridyl)-1,3,5-triazine]] and acidified with concentrated HCl and 20 mmol L^−1^ FeCl_3_. The absorbance was read at 620 nm (Multiplate reader, BMG LABTECH GmbH, SpectroStar Nano, Ortenberg, Germany). Antioxidant power was expressed as TEAC μmol 100 g^−1^ on a dry-weight basis.

2,2′-Azino-bis (3-ethylbenzothiazoline-6-sulfonic acid) (ABTS) assay was performed for leaves of var. Butternut squash (*C. moschata*) based on the method previously described by Mashitoa et al. (3) without any modifications. The ABTS cation was produced by reacting the ABTS stock solution (7 mM) with an equal volume of 4.9 mM potassium persulfate and leaving the mixture to stand in the dark at room temperature for at least 12 h before use. The ABTS+ stock solution (285 μl) was mixed with 15 μl of the 80% aqueous methanolic extract and incubated in darkness at 25°C for 2 h; subsequently, the absorbance was measured at 734 nm (BMG LABTECH GmbH, SpectroStar Nano, Ortenberg, Germany). Calibration curves were constructed using Trolox as the standard, and the antioxidant activity (ABTS assay) was expressed μmol TEAC 100 g^−1^ on a dry-weight basis.

### *In vitro* α-Amylase Inhibition Assay

The α-amylase inhibition assay was determined using porcine pancreatic α-amylase, as described previously by Moloto et al. (15), using an aliquot of 500 μl of the sample [leaf extract of var. Butternut squash (*C. moschata*)] and 500 μl of 0.02 M sodium phosphate buffer (pH 6.9), with 0.006 M sodium chloride containing α-amylase solution (0.5 mg ml^−1^). The reaction mixture was incubated at 25°C for 10 min. Thereafter, 1% starch solution (500 μl) in a 0.02-M sodium phosphate buffer (pH 6.9 with 0.006-M sodium chloride) was pipetted at different time intervals and held at 25°C for 10 min, and then the reaction was stopped by adding 100 μl of KAT amylase reagent for 5 min; the absorbance was measured at 540 nm (Multiplate reader, BMG LABTECH GmbH, SpectroStar Nano, Ortenberg, Germany). Dimethylsulfoxide (100 μl) without leaf extract and commercial α-amylase inhibitor Acarbose served as controls. The enzyme inhibitory activity was expressed as the IC_50_ (μg ml^−1^) of α-amylase inhibition.

### *In vitro* α-Glucosidase Inhibition Assay

*In vitro* α-glucosidase inhibitory activity was determined according to the method described by Mashitoa et al. (3), without any modifications, using leaf extract of var. Butternut squash (*C. moschata)* (5 μl) and concentrations of 50–250 μg ml^−1^. A 96-well plate containing 20 μl α-glucosidase solution (50 μg ml^−1^) and 60 μl of potassium phosphate buffer (pH 6.8; 67 mM) was incubated at 35°C for 5 min; 10 μl of 10-mM ρ-nitrophenyl-α-D-glucoside solution was then added and incubated at 35°C for 20 min, and after adding 25 μl of 100 mM Na_2_CO_3_, the absorbance was read at 405 nm using a microplate reader (CLARIOstar Plus BMG Labtec, Lasec, Cape Town, South Africa). The leaf extracts, acarbose, and the blank control (without α-glucosidase) were included for comparison and the IC_50_ value was measured.

### Statistical Analysis

The experiment design included a completely randomized design with 10 replicates per treatment (different drying methods) and repeated two times for accuracy. For UPLC–Q-TOF/MS and chemometric analysis, three replicate samples per drying treatment were included. A one-way ANOVA analyzed the significant differences between different postharvest drying treatments on different parameters at *p* < 0.05. Treatment means were separated using Fisher's protected least significant difference (LSD) *t*-test at the 5% level of significance. Data were analyzed using the statistical program GenStat for Windows (2004).

## Results and Discussion

### Changes in Color Properties and Pigments

Table 1 shows the influence of different drying methods with the changes in color properties. Freeze-dried leaves of var. Butternut squash (*C. moschata*) showed L^*^ (luminosity) and b^*^ (that relates to yellow) values, similar to the raw leaves. The increase in a^*^ value (higher negative), relating to the lower intensity of green color, showed the lowest color change (Δ*E* = 3.76) than the raw leaves. The highest color change (Δ*E* = 11.98) was noted in leaves that underwent microwave-drying. Table 2 shows the influence of different drying treatments on leaf pigments. Total chlorophyll content in freeze-dried leaves of var. Butternut squash (*C. moschata*) was increased by 59.24% compared with the raw leaves, followed by sun-dried (36.52%) and solar-dried (31.18%) leaves, while oven- and microwave-dried leaves showed significantly highest loss in total chlorophyll content of 16.48 and 1.11%, respectively. The higher temperatures (100°C) during oven and microwave drying could have possibly converted the chlorophyll to pheophytin, as shown in Table 1 by the olive green color and relating to higher color change (Δ*E*). Similar to the findings in this study, freeze-drying increased the total chlorophyll content in African nightshade, Chinese cabbage (11), and *Ipomoea aquatica* forsk leaves (16).

**Table 1:** Effect of different drying treatments on color values and color change in the leaves var. Butternut squash (*C. moschata*).

**Postharvest drying**	L*	a*	b*	***ΔE***
Raw	33.22 ± 2.46^c^	−7.45 ± 0.99^c^	10.61 ± 1.64^d^	
Freeze-drying	34.46 ± 0.65^c^	−4.06 ± 0.14^a^	9.54 ± 0.34^d^	3.76 ± 0.19^d^
Microwave-drying	41.71 ± 1.75^b^	−7.54 ± 0.20^c^	14.52 ± 0.73^b^	11.98 ± 0.22^a^
Oven-drying	44.41 ± 0.14^a^	−9.16 ± 0.07^bc^	16.08 ± 0.08^a^	9.83 ± 0.16^b^
Solar-drying	41.65 ± 0.84^b^	−7.38 ± 0.16^c^	12.62 ± 0.39^c^	5.70 ± 0.02^c^
Sun-drying	42.38 ± 0.85^a^	−8.66 ± 0.22^b^	13.81 ± 0.37^c^	6.13 ± 0.23^c^

*Means followed by the same letter within the column are not significantly different, p < 0.05, according to Fisher's LSD test*.

*Raw leaves data are presented on a fresh-weight basis; sun-dried leaves are included as control since they are used traditionally*.

**Table 2:** Effect of different drying treatments on chlorophylls and total carotenoids in the leaves var. Butternut squash (*C. moschata*).

**Postharvest drying**	**Total chlorophyll** ** (mgg–^**1**^)**	**% loss**	**Carotenoids** ** (mg100g–^**1**^)**	**% loss**	**Ascorbic acid content** ** (mg100g–^**1**^)**	**%loss**
Raw leaves	4.49 ± 0.52^a^		2.23 ± 0.04^a^		62.37 ± 1.86^a^	
Freeze-drying	7.15 ± 0.12^a^	59.24 ± 0.04^d^	1.01 ± 0.05^b^	−54.71 ± 1.11^c^	46.24 ± 0.92^b^	25.86 ± 0.12^e^
Microwave-drying	4.54 ± 0.13^cd^	−1.11 ± 1.25^b^	0.11 ± 0.01^d^	−95.06 ± 0.45^a^	34.41 ± 0.86^e^	44.62 ± 1.31^b^
Oven-drying	3.75 ± 0.30^d^	−16.48 ± 2.18^a^	0.08 ± 0.02^d^	−96.41 ± 2.01^a^	26.88 ± 186^f^	56.90 ± 0.43^a^
Solar-drying	5.89 ± 0.50^c^	31.18 ± 1.33^c^	0.47 ± 0.01^c^	−78.92 ± 1.65^b^	39.78 ± 1.22^c^	36.22 ± 0.11^d^
Sun-dying	6.13 ± 0.13^b^	36.52 ± 0.25^c^	0.15 ± 0.09^d^	−93.27 ± 0.77^a^	37.63 ± 0.44^d^	39.67 ± 0.71^c^

*Means followed by the same letter within the column are not significantly different, p < 0.05, according to Fisher's LSD test*.

*Sun-dried leaves are included as control since they are used traditionally*.

Conversely, different drying treatments, such as solar, sun, oven, and microwave drying, significantly increased the total chlorophyll in African nightshade and Chinese cabbage leaves compared with the raw leaves (11). More than 50% of the carotenoid content was lost during all the drying processes compared with the raw leaves. Managa et al. (11) showed, in their previous studies, that freeze-drying, solar drying, and sun drying increased the carotenoid content in African nightshade and Chinese cabbage. As per the findings in this study, oven drying and microwave drying caused significant loss of chlorophyll and carotenoid contents in African nightshade and Chinese cabbage (11). The air temperature increase relates to the loss of carotenoid content in vegetables (17). Additionally, microwave drying caused a 77.50% loss of carotenoid content in parsley leaves (18). Furthermore, freeze-drying resulted in minimum chemical changes, while oven drying at 45–140°C caused rapid degradation of primary metabolites (19).

### Ascorbic Acid Content

Freeze-dried leaves of var. Butternut squash (*C. moschata*) showed a 25.86% loss of ascorbic acid (vitamin C) compared with the raw leaves, while oven- and microwave-dried and sun-dried samples (Table 2) showed 56.90 and 44.62% loss, respectively. Besides, Gupta et al. (20) showed that ascorbic acid was better retained in freeze-dried samples than oven-dried samples. Therefore, among the tested drying methods, oven drying and microwave drying were the most unfavorable, contributing toward marked degradation of vitamin C. Managa et al. (11) reported retention of ascorbic acid in freeze-dried African nightshade and Chinese cabbage leaves compared with oven and microwave drying, as mentioned earlier. Khantoniar et al. (21). showed degradation of ascorbic acid content during microwave heating and explained that it was probably due to the power categories (90–1,000 W) during microwave drying. Furthermore, openly sun-dried cowpea leaves lost 82–86% ascorbic acid (22), whereas, in this study, sun drying caused a 39.67% loss. In this study, the solar-drying cabinet significantly reduced the loss of ascorbic acid by 36.22% in pumpkin leaves. The destruction of the cell structure during thermal drying and the contact of the oxidizing enzymes of ascorbic acid with the substrate to dehydroascorbic acid could have hydrolyzed to 2,3-diketogulonic acid and eventually polymerized to form other compounds (23, 24).

### Changes in Phenolic Metabolites

Table 3 shows the identified and quantified different phenolic components using UPLC-QTOF/MS. Gentesic acid 5-O-glucoside, 2-O-caffeoylglucaric acid, 2-O-caffeoylhydroxycitric acid, 2-(E)-O-feruloyl-D-galactaric acid isomer, 1-O-caffeoylglucose, coumaroyl glucaric acid, 2-(E)-O-feruloyl-D-galactaric acid isomer, 1-O-p-coumaroyl-beta-D-glucose, luteolin 7-neohesperidoside, 2-caffeoylisocitric acid, quercitrin, coumaroyl isocitrate, 7-methylquercetin-3-galactoside-6″-rhamnoside-3″-rhamnoside, feruloyl isocitrate, quercetin 3-galactoside 7-rhamnoside, genistin, kaempferol 7-neohesperidoside, isoorientin 2″-O-rhamnoside, isorhamnetin-3-galactoside-6″-rhamnoside, and isorhamnetin-3-O-rutinoside, pectolinarigenin 7-(6″-methylglucuronide) were identified and quantified in the leaves of var. Butternut squash belonging to *C. moschata* species (3). The impact of different drying methods on the retention of total polyphenol content in the leaves occurred in the following order: freeze-drying > solar drying > microwave drying > sun-drying > oven drying.

**Table 3:** Influence of different drying treatments on different phenolic components of leaves of var. Butternut squash (*C. moschata*).

**Phenolic components (mg kg–^**1**^)**	**Raw**	**Freeze- drying**	**Solar drying**	**Sun drying**	**Microwave drying**	**Oven drying**
2-O-caffeoylglucaric acid	89.5 ± 6.4^b^	86.3 ± 1.2^b^	117.3 ± 5.4^a^	114.7 ± 4.a	62.0 ± 12.5^c^	24.4 ± 3.3^d^
Gentesic acid 5-O-glucoside	4.8 ± 0.6^e^	48.4 ± 4.8^c^	19.9 ± 2.4^d^	82.8 ± 6.9^b^	162.0 ± 12.3^a^	11.9 ± 0.9^d^
Coumaroyl glucaric acid	55.3 ± 3.2^a^	29.9 ± 0.5^b^	28.4 ± 2.6^b^	18.7 ± 2.9^bc^	6.9 ± 0.7^c^	8.1 ± 1.5^c^
2-(E)-O-feruloyl-D-galactaric acid	83.7 ± 6.3^a^	60.4 ± 15.7^b^	49.7 ± 1.3^bc^	56.8 ± 7.3^b^	37.4 ± 4.9^c^	14.6 ± 1.9^d^
2-caffeoylisocitric acid	38.6 ± 1.9^c^	98.6 ± 1.9^a^	97.8 ± 9.3^a^	74.3 ± 4.9^b^	67.5 ± 13.3^b^	41.7 ± 3.0^c^
Coumaroyl isocitrate	47.9 ± 4.8^d^	82.2 ± 5.7^a^	64.1 ± 0.9^b^	45.8 ± 6.0^d^	51.1 ± 9.5^c^	49.4 ± 1.9^c^
7-Methylquercetin-3-Galactoside-6”-Rhamnoside-3”'-Rhamnoside;	12.3 ± 5.6^b^	17.0 ± 6.0^a^	8.3 ± 1.4^d^	15.9 ± 1.3^a^	12.7 ± 3.3^b^	0.9 ± 0.3^d^
Feruloyl isocitrate	12.6 ± 2.0^d^	44.6 ± 3.2^a^	25.9 ± 0.4^b^	32.4 ± 1.1^ab^	28.8 ± 6.6^ab^	17.0 ± 1.7^c^
Quercetin 3-galactoside 7-rhamnoside	149.9 ± 3.7^a^	111.8 ± 7.1^b^	127.9 ± 2.9^b^	63.1 ± 12.3^c^	73.9 ± 14.8^c^	19.6 ± 4.0^d^
Quercetin 3-glucoside 7-rhamnoside (Rutin)	351.9 ± 12.9^a^	354.5 ± 10.5^a^	312.9 ± 32.4^b^	256.8 ± 8.3^c^	272.3 ± 23.3^c^	94.3 ± 9.8^d^
Quercetin 3-galactoside	112.2 ± 7.7^a^	109.9 ± 1.5^a^	98.7 ± 12.3^b^	52.2 ± 3.2^c^	49.2 ± 15.0^c^	15.5 ± 4.2^d^
kaempferol 7-neohesperidoside	123.4 ± 6.4^a^	114.9 ± 5.7^ab^	96.0 ± 6.2^b^	77.6 ± 35.6^c^	117.0 ± 35.2^a^	21.1 ± 0.9^d^
Isoorientin 2”-O-rhamnoside	182.1 ± 3.7^a^	176.3 ± 3.4^a^	142.0 ± 8.3^b^	143.6 ± 10.2^b^	177.4 ± 20.2^a^	28.7 ± 2.6^c^
Isorhamnetin-3-Galactoside-6”-Rhamnoside	80.1 ± 2.1^a^	73.8 ± 9.9^a^	28.8 ± 3.0^c^	35.8 ± 11.2^b^	37.2 ± 11.3^b^	5.9 ± 0.4^d^
Isorhamnetin-3-O-rutinoside	174.1 ± 8.7^a^	174.3 ± 13.2^a^	89.6 ± 14.6^c^	125.3 ± 1.7^b^	116.6 ± 13.0^b^	14.0 ± 5.9^d^
Total polyphenolic compounds	1518.4 ± 6.9^b^	1582.9 ± 3.6^a^	1307.3 ± 5.1^c^	1195.8 ± 1.9^e^	1272.0 ± 6.8^d^	367.1 ± 3.7^f^

Freeze-dried leaves showed the highest total polyphenols compared with the raw leaves and the other four drying treatments. Freeze-dried leaves contained the highest concentration of coumaroyl isocitrate (82.20 mg kg^−1^), quercetin 3-glucoside 7-rhamnoside (Rutin) (354.50 mg kg^−1^), quercetin 3-galactoside (109.90 mg kg^−1^), isorhamnetin-3-galactoside-6″-rhamnoside (73.80 mg kg^−1^), and isorhamnetin-3-O-rutinoside (174.30 mg kg^−1^) compared with the raw leaves of var. Butternut squash and other four drying treatments. Freeze-drying enables the formation of ice crystals within the cell, and their rapid expulsion from the cell during freezing and the rapid removal of the ice crystals during the process help to maintain the cell structure that enables it to retain the phenolic compounds (25).

However, freeze-dried or microwave-dried leaves increased the concentration of feruloyl isocitrate, kaempferol 7-neohesperidoside, and isoorientin 2″-O-rhamnoside significantly in the leaves of var. Butternut squash (*C. moschata*) compared with all other drying treatments and raw leaves. Additionally, freeze-dried and sun-dried leaves of var. Butternut squash (*C. moschata*) contained the significantly highest concentration of 7-methylquercetin-3-galactoside-6″-rhamnoside-3^‴^-rhamnoside and feruloyl isocitrate compared with the other counterpart treatments and raw leaves. Oven drying negatively affected the 15 different phenolic compounds detected in the leaves of var. Butternut squash (*C. moschata*) and showed the lowest concentrations.

### Multivariate Analysis

The untargeted phenolic metabolites in the leaves of var. Butternut squash (*C. moschata*) showed differences in distribution when an unsupervised (PCA) approach was used for the data obtained by the UPLC–Q-TOF/MS analysis.

**Figure 1:**
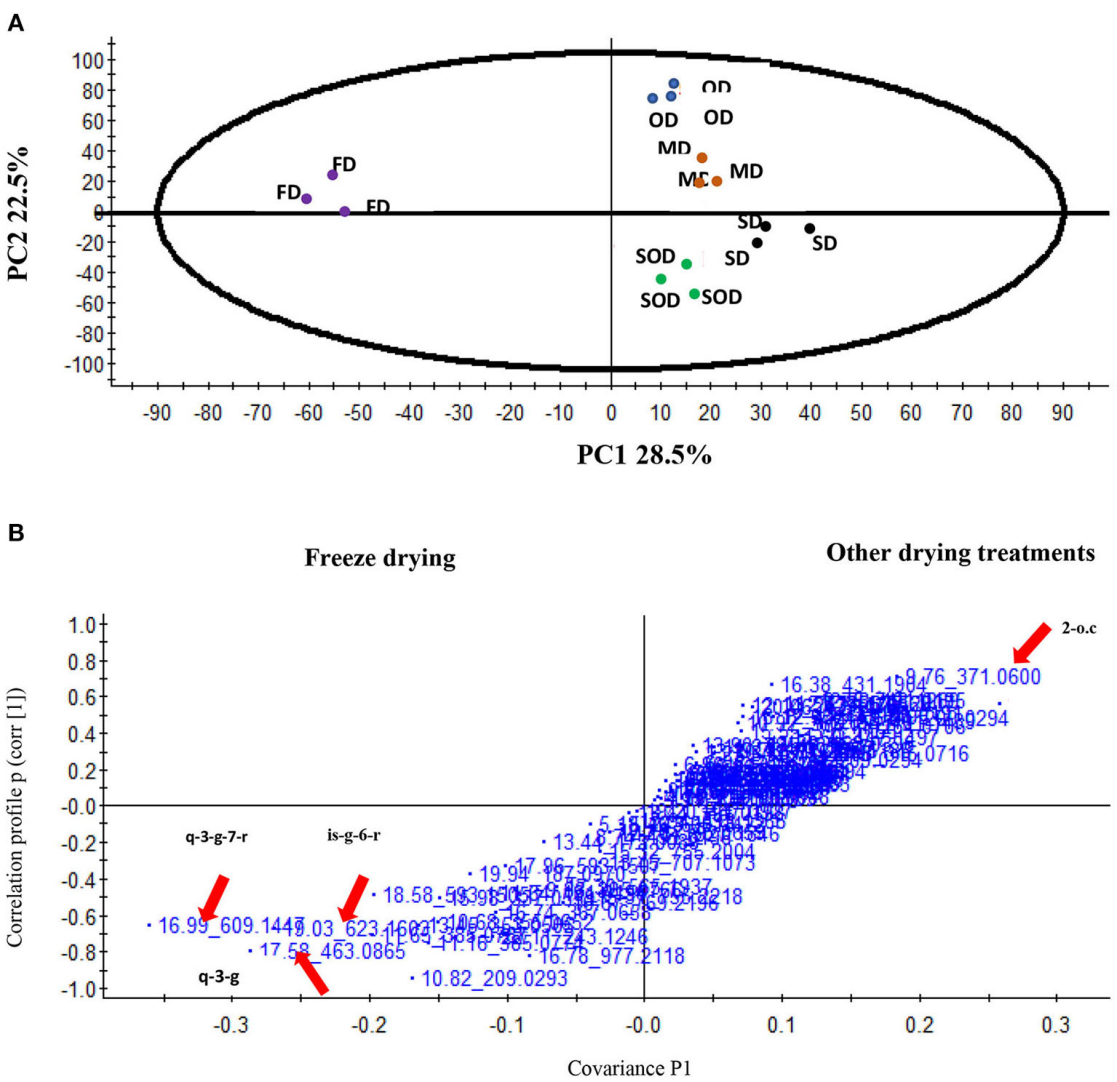
**(A)** A score plot of principal component analysis (unsupervised) based on a Waters Synapt G2 Quadrupole time-of-flight (QTOF) mass spectrometer (MS), hyphenated to a Waters Aquity ultra-performance liquid chromatograph (UPLC) (UPLC–Q-TOF/MS) spectra (phenolic metabolic analysis) of the leaves of var. Butternut squash (*Cucurbita moschata)* that underwent five different postharvest drying treatments. PC1 = 28.50% and PC2 = showing 22.5% of the leaves of three different pumpkin species. Each postharvest-drying treatment included three replicate samples. OD, oven drying; MD, microwave drying; SD, sun-drying; SOD, solar drying; FD, freeze-drying. **(B)** A score plot of orthogonal partial least squares discriminant analysis of UPLC–QTOF/MS spectra of the leaves of var. Butternut squash (*Cucurbita moschata)* subjected to different postharvest drying treatments. Each sample set includes three replicates. *m*/*z* 371.05997, 2-O-caffeoylglucaric acid (2-o.c); *m*/*z* 609.1441 quercetin 3-galactoside 7-rhamnoside (q-3-g-7- r); *m*/*z* 463.08 quercetin 3-galactoside (q-3-g); *m*/*z* 623.16 isorhamnetin-3-galactoside-6″-rhamnoside. isorhamnetin-3-galactoside-6″-rhamnoside (is-g-6-r).

The metabolite data were used to perform the PCA and discriminate the different postharvest drying treatments of var. Butternut squash (*C. moschata*) based on the greater impact of different metabolites, and PC1 accounted for 28.50% of the variance with the leaves of var. Butternut squash (*C. moschata*) that underwent freeze-drying and is positioned along with negative PC1 score values and all other drying treatments: solar, sun-, oven, and microwave drying positioned along the positive PC1 (Figure 1A). The phenolic metabolites that showed a greater impact on PC2 accounted for 22.50% of the variance with the leaves of var. Butternut squash (*C. moschata*) that underwent microwave drying positioned along with positive PC2 and the rest of the drying treatments along the negative PC2 (Figure 1A) based on the metabolites, facilitating the discrimination of the leaves of var. Butternut squash (*C. moschata*) that underwent different kinds of postharvest drying. OPLS-DA was performed as a tool for metabolite data to analyze the multivariate data to relate a quantitative relationship between leaves of var. Butternut squash (*C. moschata*), and the four different kinds of postharvest drying and the phenolic compounds helped to identify the marker candidate responsible for the separation of the four different drying treatments. Figure 1B, Table 5 illustrates the S-plot, showing the marker ions *m*/*z* 609,14 (quercetin 3-galactoside 7-rhamnoside), *m*/*z* 463.08 (quercetin 3-galactoside), *m*/*z* 623.16 (isorhamnetin-3-galactoside-6″-rhamnoside) separating the freeze-dried leaves of var. Butternut squash (*C. moschata*) from the other four drying treatments, while the other four drying treatments were separated from the freeze-drying by the marker ion *m*/*z* 371,06 (2-O-caffeoylglucaric acid).

### Antioxidant Activities and Inhibitory Effect of α-Amylase and α-Glucosidase

Freeze-dried leaves of var. Butternut squash (*C. moschata*) showed the highest FRAP (383.13 μmol TEAC100 g^−1^) and ABTS (25.01 μmol TEAC100 g^−1^) activities, while the oven-dried samples showed the lowest antioxidant activities, probably due to the lower polyphenol content, as shown in Table 4. Similarly, Managa et al. (11) showed higher FRAP and ABTS activities in the leaves of freeze-dried African nightshade and Chinese cabbage and the lowest antioxidant activity in oven-dried leaves. The antioxidant property of the vegetables depends on the presence and the concentrations of antioxidant components, and thermal processing at 65 or 100°C reduced the antioxidant activities in food products (26).

**Table 4:** Effect of different drying treatments on antioxidant activities and inhibitory effect on digestive enzymes in the leaves var. Butternut squash (*C. moschata*).

**Treatments**	**FRAP** ** (μmol TEAC 100 g^**−1**^)**	**ABTS** ** (μmo lTEAC 100 g^**−1**^)**	**α-Glucosidase** ** IC_**50**_ (μgmL^**−1**^)**	**α-Amylase** ** IC_**50**_ (μgmL^**−1**^)**
Raw	141.88 ± 0.36^b^	23.35 ± 0.80^c^	21.20 ± 0.21	18.11 ± 0.11^c^
Freeze dried	383.13 ± 0.30^a^	25.01 ± 1.1^a^	19.14 ± 0.13^e^	16.97 ± 0.14^e^
Microwaved dried	233.33 ± 0.21^de^	22.04 ± 1.2^d^	26.09 ± 0.12^b^	20.34 ± 0.08^a^
Oven dried	141.41 ± 0.01^e^	10.18 ± 0.1.01^e^	27.24 ± 0.10^a^	23.44 ± 0.13^a^
Solar dried	242.42 ± 0.14^cd^	24.31 ± 1.1^b^	22.39 ± 0.10^d^	17.14 ± 0.04^d^
Sun dried	248.16 ± 0.02^c^	23.52 ± 0.90^c^	25.20 ± 0.17^c^	18.83 ± 0.06^c^
			Acarbose 18.03 ± 0.11^f^	Acarbose 19.07 ± 0.01^b^

*Means followed by the same letter within the column are not significantly different, p < 0.05, according to Fisher's LSD test*.

*Sun-dried leaves are included as control since it is used traditionally*.

**Table 5:** Exact mass/retention time pairs responsible for the separation of freeze-dried pumpkin leaves from the other drying treatments.

**Primary ID**	**Retention time**	**Mass**	**p[1]P**	**p(corr)[1]P**	**Factor of change**	**Freeze drying**	**Others**
Quercetin 3-galactoside	17.58	463.08	−0.28	−0.79	1.9	279.07	537.61
Isorhamnetin-3-galactoside-6″-rhamnoside	19.03	623.16	−0.26	−0.66	2.0	417.64	207.73
Quercetin 3-galactoside 7-rhamnoside	16.99	609.14	−0.36	−0.66	2.1	541.08	263.03
2-O-caffeoylglucaric acid	6.963	371.05	0.24	0.68	1.4	1337.7	945.08

Freeze-drying is a nonthermal process, which probably conserved the antioxidants, such as ascorbic acid and carotenoids, of pumpkin leaves. These compounds have higher redox potential and contribute significantly toward antioxidant activities (11, 26). In addition, the presence of higher numbers of hydroxyl groups in phenolic compounds contributes more toward the antioxidant activity (26). The leaves of var. Butternut squash (*C. moschata*) contain flavonoid glycosides, and, sometimes, the presence of mono- or diglycosidic molecules and the arrangement of hydroxyl group on the flavonoid B and C rings affect the antioxidant property. Furthermore, in the sun-, solar-, and oven-dried leaves, the thermal process could have released the antioxidants due to destruction of the cell wall and cellular components or production of antioxidants, such as Millard-derived melanoidins (27), and denaturation of polyphenol oxidase and polyphenol peroxidase enzymes could have protected the antioxidants and their activity (13). The microwave output power also plays a vital role in determining the antioxidant activity in vegetables, and the higher output power of 800 W improves the antioxidant activity (28). Phenolic antioxidants correlated well with FRAP activity (29). In this study, FRAP activity correlated strongly with quercetin 3-glucoside 7-rhamnoside (rutin) (*r* = 0.80, *p* < 0.05), coumaroyl isocitrate (*r* = 0.75, *p* < 0.05) and feruloyl isocitrate (*r* = 0.73, *p* < 0.05). Therefore, higher retention of polyphenolic compounds and antioxidant activity in freeze-dried leaves of var. Butternut squash (*C. moschata*) acts as a good natural antioxidant, a food preservative, or a dietary supplement in health-promoting foods.

Table 4 presents the inhibition of α-glucosidase and α-amylase activities. Inhibitory activities of α-glucosidase (IC_50_ 19.77 μgml^−1^) and α-amylase (IC _50_ 16.97 μgml^−1^) were highest in freeze-dried leaves of var. Butternut squash with sucrose substrate compared with the other four postharvest drying treatments and raw leaves. However, oven-dried leaves reduced the inhibition of α-glucosidase and α-amylase compared with the other drying treatments and raw leaves. The acarbose showed the significantly highest inhibition of α-glucosidase activity (IC_50_18.03 μgml^−1^) compared with the freeze-dried leaf extract samples. At the same time, acarbose showed a lower inhibitory effect on α-amylase (IC_50_ 19.07 μgml^−1^) than the freeze-dried leaf extract.

The presence of the hydroxyl group on position 4 (ring B) of the molecular structure of quercetin 3 galactoside, isorhamnetin-3-galactoside-6″-rhamnoside, isoorientin 2″-O-rhamnoside, genistin, quecetin 3 galactoside 7-rhamnoside, kaempferol neohesperoside, isohermnetin 3-O-rutinosidem, and rutin plays a vital role in their α-glucosidase and α-amylase inhibitory effect (30, 31). Furthermore, quercetin 3 galactoside, isorhamnetin-3-galactoside-6″-rhamnoside, isoorientin 2″-O-rhamnoside have another hydroxyl group in position 7 (ring A), which also contributes to the α-glucosidase and α-amylase inhibitory effect (30). Besides, the C2 = C3 double bond in the C ring of flavonoids, such as quercetin 3 galactoside, isorhamnetin-3-galactoside 6" rhamnoside, and isoorientin 2" rhamnoside, is essential for their α-glucosidase and α-amylase inhibition activity (31). The degree of high hydroxylation of the non-flavonoid phenolic compounds, including gentesic acid, 2 caffeoylglucaric acid, 2 caffeoyl hydroxycitric acid, feruloyl isocitrate, and 1-O-caffeoyl glucose, also could have significantly enhanced their amylase inhibition activity (30). Furthermore, the synergistic effect on different phenolic compounds and their varying concentrations could have had a great impact on the degree of inhibition of these two digestive enzymes.

Although the Food and Drug Administration has approved commercial synthetic-inhibiting agents, the synthetic inhibitor caused side effects, e.g., liver disorders. Therefore, plant-based enzyme inhibitors, such as the leaf extracts of var. Butternut squash (*C. moschata*), can be regarded as safer to manage type 2 diabetics (32).

## Conclusions

The presented data indicated that freeze-drying was the best postharvest drying method. Freeze-drying preserved the color properties, most phenolic metabolites, antioxidant activity, and antidiabetic activity of the leaves of var. Butternut squash (*C. moschata*). Oven-drying caused the highest reduction of different phenolic metabolites, ascorbic acid content, antioxidant activity, and inhibitory activities of α-glucosidase and α-amylase. The study also helped to identify the biomarker candidates that separated the freeze-dried pumpkin leaves from the samples that underwent other drying treatments using novel practical tools, such as UPLC-QTOF/MS and the chemometric approach. Although freeze-drying is expensive at the rural level, industrial and pilot-scale freeze dryers are available for the production of functional powders in large-scale at privatelyowned companies involved in the functional powder trade. Therefore, the findings of this study provide evidence-based information that the use of an appropriate postharvest drying method to obtain the functional powder from the leaves of var. Butternut squash (*C. moschata)* is a functional ingredient for the further development of functional foods.

## Data Availability Statement

The original contributions generated for the study are included in the article/Supplementary Material, further inquiries can be directed to the corresponding author/s.

## Author Contributions

FM was the first author and the Ph.D. student who performed the experiment, generated the data, and wrote some parts of this manuscript. TS visualized and validated the data for phenolic compounds, interpreted the chromatogram, and wrote that part of the article. JS was responsible for the antidiabetic activity and data. RS, the research collaborator and co-supervisor, provided editorial support. YS the research collaborator, presented the data visualization. DS the grant holder, conceptualized the research, supervised the first author, and improved the article further. All authors contributed to the article and approved the submitted version.

## Conflict of Interest

The authors declare that the research was conducted in the absence of any commercial or financial relationships that could be construed as a potential conflict of interest.
