# Supplementary material for: Influence of Different Types of Drying Methods on Color Properties, Phenolic Metabolites and Bioactivities of Pumpkin Leaves of var. Butternut squash (Cucurbita moschata Duchesne ex Poir)
Source: Front Nutr. 2021 Jun 29;8:694649. doi: 10.3389/fnut.2021.694649 (PMC8275642; doi:10.3389/fnut.2021.694649)
Supplement: Supplementary file 1 [file Data_Sheet_1.pdf]

Supplementary Table 1 Tentative peak assignment of the phenolic metabolites present in leaves of var. Butternut squash (*C. moschata*) subjected to different drying treatments.

	Retention time (min)	[M-H]$^-$	M-H formula	Error (ppm)	MSE fragments	UV	Tentative Identification
	8.03	315.0720	$C_{13}H_{15}O_9$	0.32	108.0212<br>151.9972	314	Gentesic acid 5-O-glucoside
	8.12	371.0605	$C_{15}H_{15}O_{11}$	4.04	191.0259<br>209.0361	323	2-O-caffeoylglucaric acid
	10.69	355.0667	$C_{15}H_{15}O_{10}$	1.12	163.0501<br>209.0332,	314	Coumaroyl glucaric acid
	11.15	385.0772	$C_{16}H_{17}O_{11}$	1.04	85.02256<br>385.0714	320	2-(E)-O-feruloyl-D-galactaric acid isomer
1	13.45	353.0502	$C_{15}H_{13}O_{10}$	3.40	191.0261	331	2-caffeoylisocitric acid
	15.63	337.0552	$C_{15}H_{13}O_9$	3.86	119.0431<br>191.0261	314	Coumaroyl isocitrate
	16.67	769.2119	$C_{23}H_{45}O_{28}$	-2.08	300.0241<br>315.0345	330	7-Methylquercetin-3-Galactoside-6''-Rhamnoside-3'''-Rhamnoside
	16.74	367.0648	$C_{16}H_{15}O_{10}$	6.27	173.0066<br>111.0049	329	Feruloyl isocitrate
	16.80	609.1441	$C_{27}H_{29}O_{16}$	3.28	147.0174<br>151.0100<br>162.9927<br>179.0179<br>300.0256<br>272.5696	255	Quercetin 3-galactoside 7-rhamnoside
18	17.00	609.1444	$C_{27}H_{29}O_{16}$	2.79	151.0011<br>178.9964<br>273.0332	255	Quercetin 3-glucoside 7-rhamnoside (Rutin)

					300.0256		
20	17.96	593.1526	$C_{27}H_{29}O_{15}$	-2.36	228.0345<br>256.0335<br>285.0390	264	Kaempferol 7-neohesperidoside
21	18.59	593.1513	$C_{27}H_{29}O_{15}$	-0.17	120.0294<br>447.0653<br>473.1074	265	Isoorientin 2"-O-rhamnoside
22	18.83	623.1610	$C_{28}H_{31}O_{16}$	1.77	299.0212<br>315.0538	265	Isorhamnetin-3-Galactoside-6"-Rhamnoside
23	19.03	623.1600	$C_{28}H_{31}O_{16}$	3.37	299.0239<br>315.0662	255	Isorhamnetin-3-O-rutinoside
