# Supplementary material for: Influence of Different Types of Drying Methods on Color Properties, Phenolic Metabolites and Bioactivities of Pumpkin Leaves of var. Butternut squash (Cucurbita moschata Duchesne ex Poir)
Source: Front Nutr. 2021 Jun 29;8:694649. doi: 10.3389/fnut.2021.694649 (PMC8275642; doi:10.3389/fnut.2021.694649)
Supplement: Supplementary file 2 [file Data_Sheet_2.pdf]

	Name	Sample Text	Catechin Response	Epicatechin Response	Rutin Response
1	DS_TUT_200813_51	Blank	0,269	-	30,84
2	DS_TUT_200813_52	2.5 ppm phenolics	184,811	230,878	206,517
3	DS_TUT_200813_53	5 ppm phenolics	321,852	393,543	334,931
4	DS_TUT_200813_54	10ppm phenolics	590,982	816,574	581,193
5	DS_TUT_200813_55	25 ppm phenolics	1374,29	1611,743	1186,428
6	DS_TUT_200813_56	50 ppm phenolics	2719,897	2973,184	2253,722

Compound name: catechin
Correlation coefficient: r = 0.999972, r^2 = 0.999945
Calibration curve: 53.2446 * x + 53.3416
Response type: External Std, Area
Curve type: Linear, Origin: Exclude, Weighting: 1/x, Axis trans: None

Compound name: epicatechin
Correlation coefficient: r = 0.995703, r^2 = 0.991425
Calibration curve: 59.5242 * x + 103.986
Response type: External Std, Area
Curve type: Linear, Origin: Exclude, Weighting: 1/x, Axis trans: None

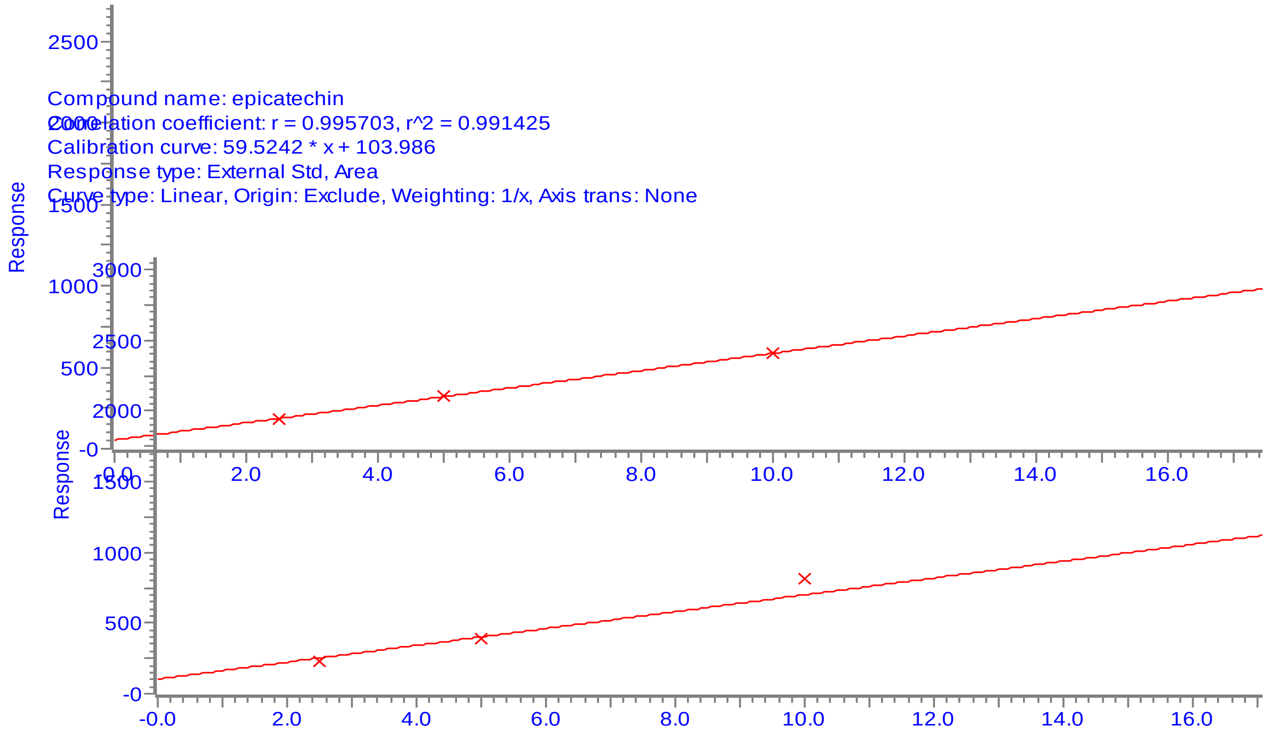

Compound name: Rutin
Correlation coefficient: r = 0.999000, r^2 = 0.998002
Calibration curve: 43.4106 * x + 109.461
Response type: External Std, Area
Curve type: Linear, Origin: Exclude, Weighting: 1/x, Axis trans: None

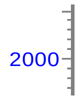

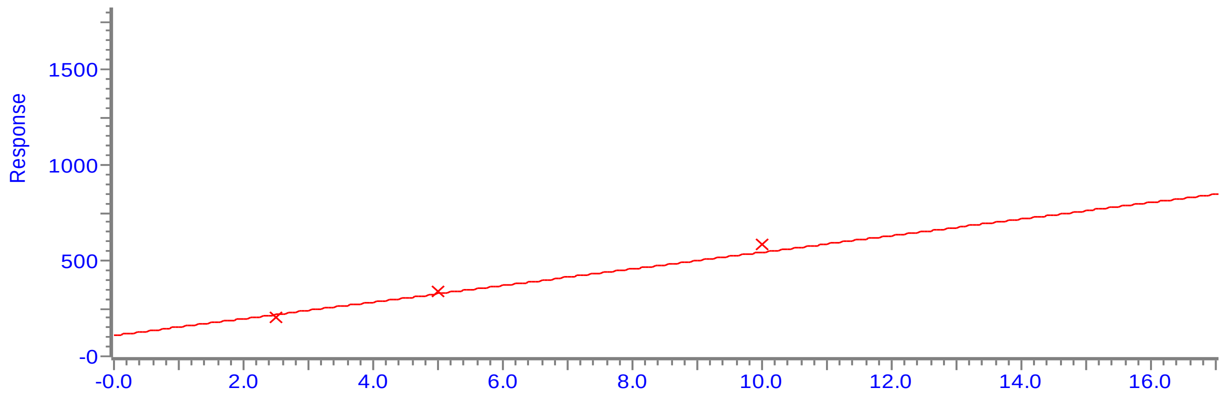

Supplementary Figure 1 . Illustrating the quatifications of compounds using the reference

Catechin	
conc X	abs Y
0	0,269
2,5	184,811
5	321,852
10	590,982
25	1374,29
50	2719,897
LOD	3,3*(Sb/a)
LOQ	10*(Sb/a)
Steyx (Sb)	23,01649
Slope (a)	53,70335
LOD	1,414333
LOQ	4,285857

Epicatechin	
ConcX	Abs Y
0	0
2,5	230,878
5	393,543
10	816,574
25	1611,743
50	2973,184
LOD	3,3*(Sb/a)
LOQ	10*(Sb/a)
Sb	90,23955
a	58,33423
LOD	5,104902
LOQ	15,4694

Conc X	
0	
2,5	
5	
10	
25	
50	
LOD	
LOQ	
Sb	
a	
LOD	
LOQ	

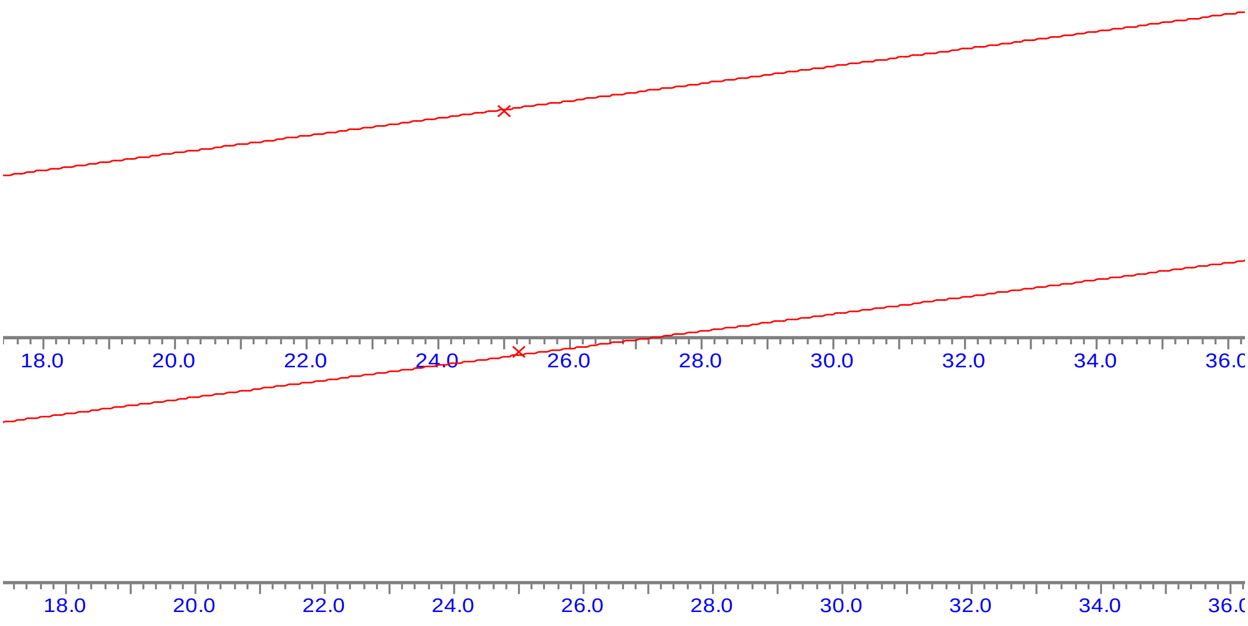

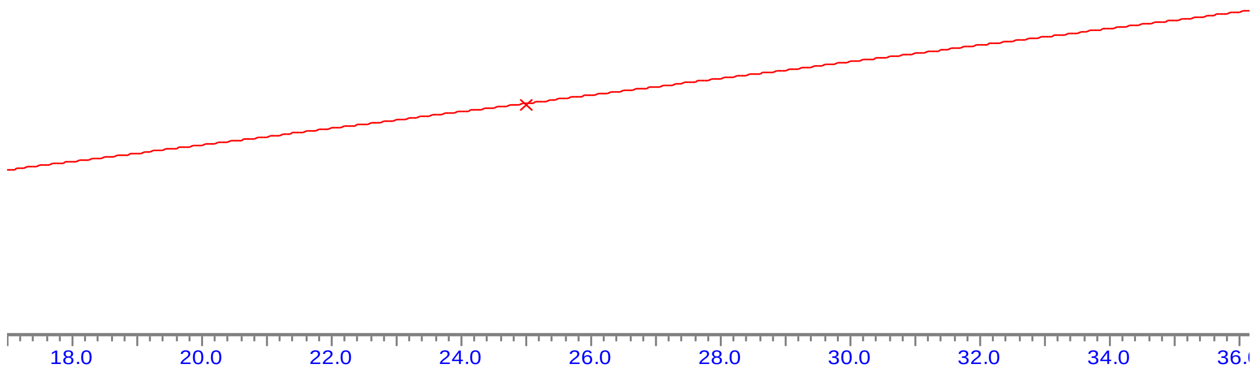
18.0
20.0
22.0
24.0
26.0
28.0
30.0
32.0
34.0

RUTIN
Abs Y
30,84
206,517
334,931
581,193
1186,428
2253,722
3,3*(Sb/a)
10*(Sb/a)

43,37247
43,45472

3,293753
9,98107

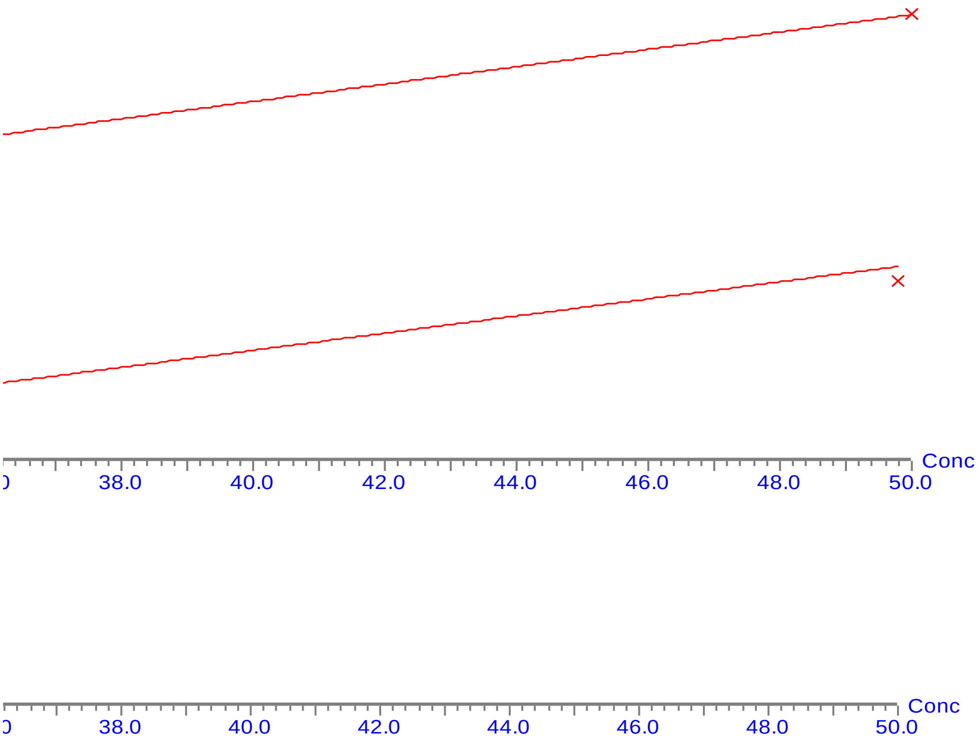

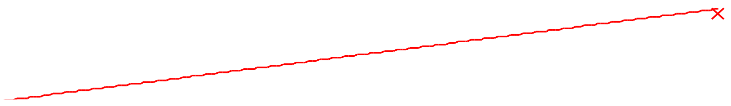

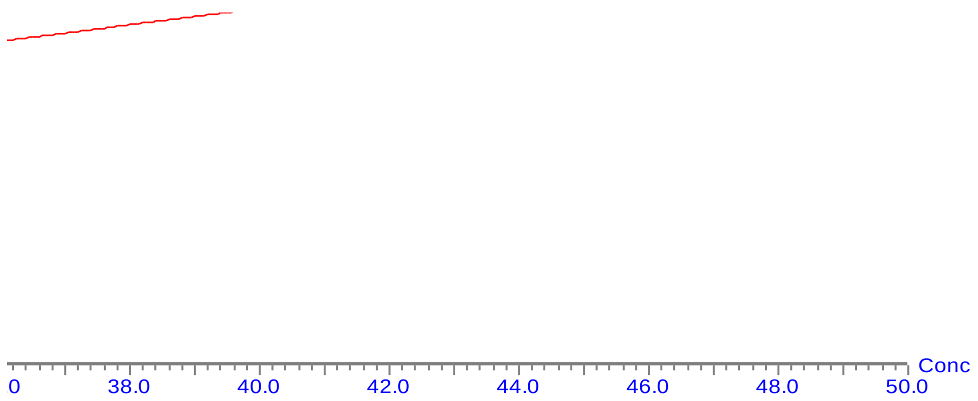
Conc
0
38.0
40.0
42.0
44.0
46.0
48.0
50.0
